# The Co-Existence of *mcr-1.1* and *mcr-3.5* in *Escherichia coli* Isolated from Clinical Samples in Thailand

**DOI:** 10.3390/antibiotics14060596

**Published:** 2025-06-10

**Authors:** Panida Nobthai, Sirigade Ruekit, Dutsadee Peerapongpaisarn, Prawet Sukhchat, Brett E. Swierczewski, Nattaya Ruamsap, Paphavee Lertsethtakarn

**Affiliations:** 1 Department of Bacterial and Parasitic Diseases, Walter Reed Army Institute of Research-Armed Forces Research Institute of Medical Sciences (WRAIR-AFRIMS), Bangkok 10400, Thailand; panidan@afrims.org (P.N.); sirigader@afrims.org (S.R.); dutsadeep@afrims.org (D.P.); nattayar@afrims.org (N.R.); 2 Queen Sirikit Naval Hospital, Chonburi 20180, Thailand; psukhchat@gmail.com; 3 US Army Medical Research Institute of Infectious Diseases, Frederick, MD 21702, USA; brett.e.swierczewski.mil@health.mil

**Keywords:** coexistence of drug resistance plasmids, colistin resistance, *mcr* genes

## Abstract

The emergence of colistin resistance poses a significant threat to its efficacy as a last-line treatment against multidrug-resistant Gram-negative bacterial infections. In this study, 178 multi-drug resistant (MDR) *Escherichia coli* isolates collected from clinical samples at Queen Sirikit Naval Hospital, Chonburi, Thailand, were evaluated for colistin resistance. Of these, six were identified as *mcr* gene carriers, mediating colistin resistance. Specifically, *mcr-1* was detected in three *E. coli* isolates, *mcr-3* was detected in one *E. coli* isolate, and *mcr-1* and *mcr-3* were detected in two *E. coli* isolates, designated AMR-0220 and AMR-0361. Whole-genome sequencing and bioinformatics analysis revealed that AMR-0220 and AMR-0361 belonged to ST410 and ST617 lineages, respectively. Both isolates carried multiple plasmids, with *mcr-1.1* located on an IncX4-type plasmid that is closely related to previously reported *mcr-1.1*-carrying IncX4 plasmids. In contrast, *mcr-3.5* was identified on distinct plasmid backbones: an IncFIB-type plasmid in AMR-0220 and an IncFII-type plasmid in AMR-0361. Overall, our findings demonstrate that the *mcr* genes found in *E. coli* isolates in this region are located on different mobile genetic elements, indicating the potential for a widespread dissemination of colistin resistance among Gram-negative bacteria throughout Thailand’s healthcare system.

## 1. Introduction

Antimicrobial resistance (AMR) in *Enterobacteriaceae* has become a global health concern. One of the main causes of increasing AMR is the overuse of antibiotics coupled with the lack of development of new antibiotics [1]. Older drugs, such as colistin, are considered last-line antibiotics for the treatment of infections caused by multidrug-resistant (MDR) pathogens that are resistant to three or more different antimicrobial drug classes. However, the overuse or misuse of colistin among humans and animals has led to the emergence of colistin-resistant pathogens [2]. Southeast Asia, including Thailand, is recognized as a global hotspot for AMR [3]. Between 2020 and 2022, resistance to carbapenems and third-generation cephalosporin in clinical *E. coli* isolates declined, while colistin resistance notably increased from 5.8% to 19.9%, highlighting a growing public health concern in Thailand [4]. Since the first report of the plasmid-mediated mobilized colistin-resistant gene (*mcr-1*) among *Escherichia coli* (*E. coli*) isolates in China in late 2015 [5], several other *mcr* genes (*mcr-2* to *mcr-10*) and their variants have been reported worldwide [6]. As of October 2024, 37 *mcr-1* variants have been submitted to the NCBI National Database of Antibiotic Resistant Organisms (NDARO) (https://www.ncbi.nlm.nih.gov/pathogens/antimicrobial-resistance/, accessed on 24 October 2024), while *mcr-1* has remained prevalent and continues to be detected worldwide in various bacterial species and sources.

In Thailand, the first report of *mcr-1* was isolated from *E. coli* in an asymptomatic individual in 2016 [7]. To date, seven *mcr* genes (*mcr-1*, *mcr-2*, *mcr-3*, and *mcr-6* to *mcr-9*) have been detected in *Enterobacteriaceae* isolated from animal and human sources [7,8,9,10]. Although uncommon, the co-occurrence of different *mcr* genes within a single bacterial isolate has been recently reported in *E. coli* from clinical samples in China [11] and New Zealand [12], and from livestock in China [13], Spain [14], and France [15]. In Thailand, the co-existence of *mcr* genes has been reported in *Klebsiella pneumoniae* (*K. pneumoniae*) from a healthy individual [16] and in *E. coli* from both healthy individuals and livestock [17]. However, the co-occurrence has rarely been reported in *E. coli* from clinical samples. This study reports the genomic characterization of antimicrobial resistance genes and susceptibility profiles of six colistin-resistant *E. coli* isolated from clinical samples in Thailand. Notably, two clinical *E. coli* isolates, AMR-0220 and AMR-0361, were found to co-carry *mcr-1.1* and *mcr-3.5* genes on distinct plasmids. This co-occurrence in clinical isolates highlights the risk of enhanced resistance and plasmid-mediated spread, underscoring the need for ongoing surveillance in healthcare settings.

## 2. Results and Discussion

Of the 178 MDR *E. coli* isolates, six colistin-resistant isolates (AMR-0201, AMR-0220, AMR-0251, AMR-0354, AMR-0361, and AMR-0429) were identified and were characterized further. These six isolates were susceptible to carbapenems, nitrofurantoin, and piperacillin–tazobactam. Five isolates were intermediately resistant to tobramycin, whereas one isolate was susceptible (Table 1). All were resistant to colistin, amoxicillin–clavulanate, ampicillin, aztreonam, and tested cephems; however, resistance to cefoxitin was identified in only one isolate. Three isolates were resistant to ciprofloxacin, gentamicin, norfloxacin, trimethoprim, and trimethoprim–sulfamethoxazole (Table 1).

The six colistin-resistant *E. coli* isolates were initially screened for ESBL, carbapenemase, and colistin resistance genes (*mcr*-1 to *mcr*-9) using conventional and real-time PCR assays. Each isolate was found to carry at least one *mcr* gene, together with one or more β-lactamase genes, including *bla*_TEM_, *bla*_CMY_, or *bla*_CTX-M_. Specifically, the *mcr-1* gene was identified in three isolates (AMR-0201, AMR-0251, and AMR-0354), the *mcr-3* gene was identified in one isolate (AMR-0429), and the coexistence of the *mcr-1* and *mcr-3* genes was identified in two isolates (AMR-0220 and AMR-0361). None of the isolates carried carbapenemase genes (*bla*_KPC_, *bla*_NDM_, *bla*_OXA-48-like_, *bla*_IMP_, and *bla*_VIM_).

To further investigate the molecular characteristics of six *mcr*-carrying *E. coli* isolates, whole-genome sequencing (WGS) was performed using Illumina short-read sequencing. In addition, two isolates (AMR-0220 and AMR-0361) carrying both *mcr-1* and *mcr-3* were subjected to long-read sequencing using the PacBio RS II platform. The sequencing results revealed the presence of acquired resistance genes, including *mcr-1.1*, *mcr-3.4*, or coexistence of *mcr-1.1* and *mcr-3.5*, all of which related to colistin resistance (Table 2). Furthermore, all isolates harbored genes for beta-lactam antibiotic resistance, with *bla*_CTX-M-55_ found in all isolates. In addition, *bla*_TEM-1_ was identified in three isolates, while *bla*_CTX-M-15_ and *bla*_CMY-2_ were identified in one isolate each. Genes conferring resistance to aminoglycosides, phenicols, and sulfonamides were present in five isolates, whereas AMR-0354 isolate lacked AMR genes from these categories. Additional AMR genes, conferring resistance to quinolone, macrolide, lincosamide, tetracycline, and rifampin, were also identified, as shown in Table 2.

In silico multilocus sequence typing (MLST) revealed five distinct sequence types (STs): ST410 (*n* = 2), ST10, ST38, ST46, and ST617 (Table 2). According to a previous study, ST410 has been reported worldwide as an MDR lineage that causes intestinal, urinary tract, and bloodstream infections in humans [18]. We found that isolates AMR-0201 and AMR-0220 both belonged to the ST410 lineage and carried genes associated with resistance to nine antibiotic classes (Table 2). Notably, ST410 is one of the most frequently identified sequence types among clinical *E. coli*-harboring *mcr* genes reported across Asia, Europe, and the Americas [19]. AMR-0251 and AMR-0361 belong to ST10 and ST617, respectively. Both isolates are members of the ST10 complex, which has been reported as the predominant lineage among clinical *mcr*-positive *E. coli*, particularly in Asia and Europe [19]. These isolates also carried genes associated with resistance to more than seven antibiotic classes. The whole-genome sequences were deposited in GenBank under BioProject No. PRJNA814829 [20].

**Figure 1:**
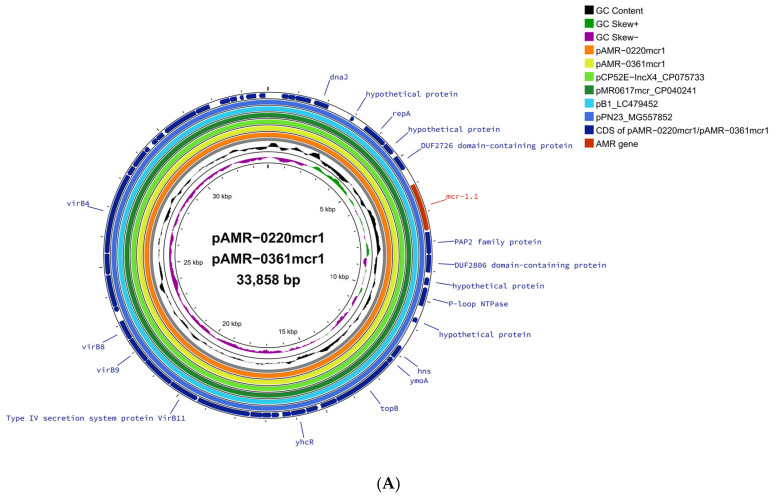

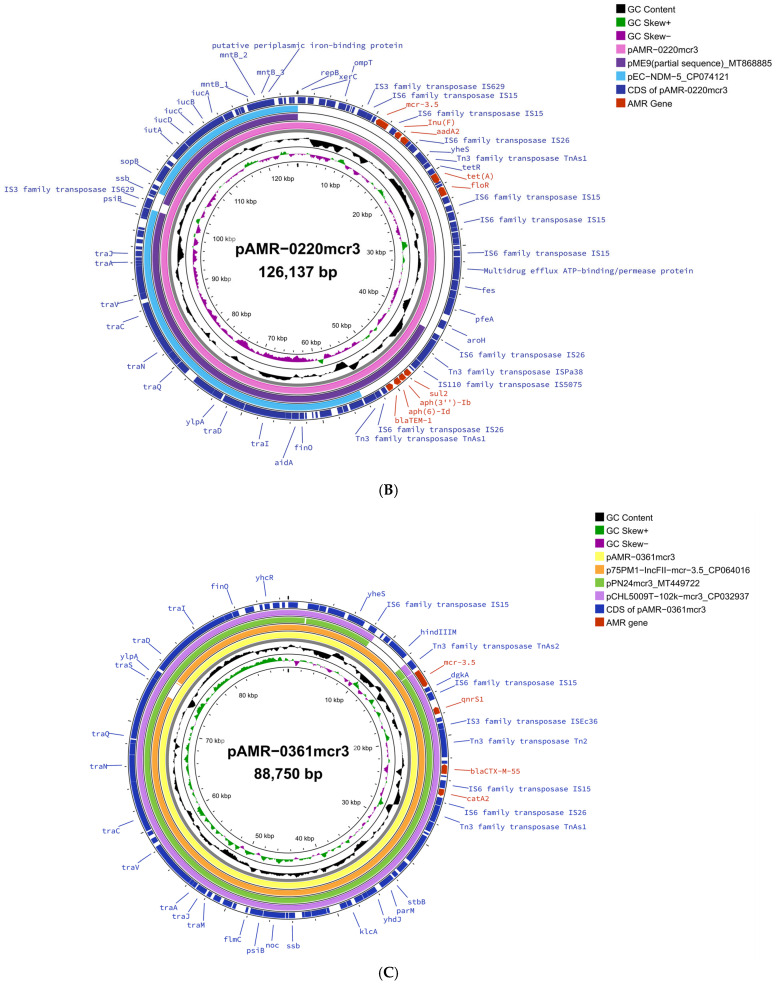
Comparison between *mcr*-carrying plasmids to the most similar plasmid deposited in the NCBI database, constructed using CGView (http://proksee.ca, accessed on 24 October 2024). (**A**) pAMR-0220mcr1 and pAMR-0361mcr1, (**B**) pAMR-0220mcr3, and (**C**) pAMR-0361mcr3. The GC skew (green and magenta), and GC content (black) of the plasmids are represented in the inner circles. The blue arrows represent CDSs and red arrows represent resistance genes.

Comparative analysis of the *mcr-1.1*-carrying plasmid pAMR-0220mcr1 and pAMR-0361mcr1 to reference plasmids showed that there was 100% nucleotide identity and 100% coverage with the following plasmids: pMR0617mcr (GenBank accession no. CP024041) of *K. pneumoniae* QS17-0029 cultured from a clinical sample from Thailand [21], pCP52E-Inc-X4 (GenBank accession no. CP075733) of *E. coli* isolated from healthy humans in Thailand [22], pB1 (GenBank accession no. LC479452) of *E. coli* B1 isolated from a wastewater sample from Japan [23], and pPN23 (GenBank accession no. MG557852) of *E. coli* PN23 isolated from a duck in Thailand [24], as shown in Figure 1A. The IncX4 plasmid is reported to be one of the most common plasmids carrying the *mcr-1* gene, as it has been identified globally including in Thailand among human isolates [25,26]. The sequence of pAMR-0361mcr1 and pAMR-0220mcr1 was identical to pAMR0617mcr, which was carried by a *K. pneumoniae* isolate that we previously isolated from different clinical samples in the same hospital in 2017 [21]. These results suggest that it is possible that this IncX4-*mcr-1.1* plasmid has been circulating in *Enterobacteriaceae* among patients in this hospital for some time, likely driven by horizontal gene transfer. The detection of an identical plasmid across different isolates raises public health concerns, as it may facilitate the silent spread of colistin resistance within healthcare settings and beyond.

pAMR-0220mcr3 was identified as a ~126 kb IncFIB plasmid carrying *mcr-3.5* based on BLAST analysis (BLASTN V2.16.0). As of Oct 2024, no exact match was found in the NCBI database (Figure 1B). The sequence showed high similarity but with low coverage when compared to reference plasmids pME9 (GenBank accession no. MT868885) and pEC16-NDM-5 (GenBank accession no. CP074121). Notably, neither of those reference plasmids contained the *mcr-3.5* gene. pAMR-0220mcr3 exhibited over 99% similarity with 61% coverage to the pME9 plasmid of *E. coli* isolated from a water source in France, which harbored four similar resistance genes, *aph(3″)-Ib*, *aph(6)-Id*, *sul2*, and *bla*_TEM-1B_, to pAMR-0220mcr3. However, since pME9 was deposited in Genbank as a partial sequence, the actual identity and coverage could not be further investigated. pEC16-NDM-5 was carried by an *E. coli* isolate from a patient in China [27]. pAMR-0220mcr3 showed 48% sequence coverage and over 99% identity with pEC19-NDM-5 (~146 kb). However, resistance genes on pEC16-NMD-5 including *bla*_NDM-5_, *dfrA12*, *aadA2*, *sul1*, *aac(3)-IV,* and *aph(4)-Ia* were not found on pAMR-0220mcr3. The AMR genes found on pAMR-0220mcr3 were *aadA2*, *aph(3”)-Ib*, *aph(6)-Id*, *bla*_TEM-1B_, *floR*, *Inu(F)*, *mcr-3.5*, *sul2*, and *tet(A)* (Figure 1B).

The IncFII plasmid, pAMR-0361mcr3 (~89 kb), showed >99% nucleotide similarity with ≥95% coverage when compared to other IncFII plasmids, including p75M1-IncFII-mcr-3.5 (GenBank accession no. CP064016), which carries *mcr-3.5*; pCHL5009T-102k-mcr3 (GenBank accession no. CP032937); and pPN24 (GenBank accession no. MT449722), both carrying *mcr-3.1* (Figure 1C). A previous study reported that IncFII was one of the predominant plasmids that carried *mcr-3* [28]. These four IncFII plasmids shared a common plasmid backbone (>95% identity) with gene-encoding resistance to colistin (*mcr-3*), quinolone (*qnrS1*), beta-lactam (*bla*_CTX-M-55_), and chloramphenicol (*catA2*). p75M1-IncFII-mcr-3.5 (~88 kb) was detected in *E. coli* isolated from a farm pig in Thailand in 2023 [17]. pCHL5009T-102k-mcr3 (~102 kb) was detected in *E. coli* that was isolated from a patient in New Zealand who had traveled to Thailand prior to a hospitalization in 2017 [12]. pPN24 (~97 kb) was detected in *E. coli* that was isolated from a duck in Thailand in 2021 [29]. Of note, pPN24 was previously reported to harbor *mcr-1* in an *E. coli* isolate by Yang Q. et al. [24], but was later found to also harbor *mcr-3.1* by Tansawai U. et al. [29]. These findings suggest that IncFII-type plasmids may play an important role in the transmission of *mcr-3* variants between human and animal sources. The IncF plasmid family, including IncFII, is a significant contributor for the dissemination of antibiotic resistance genes, especially ESBL genes, genes encoding carbapenemase, genes encoding aminoglycoside-modifying enzymes, and plasmid-mediated quinolone resistance (PMQR) genes in *E. coli* [30]. *mcr-3*-carrying IncFII plasmids have been reported to transfer efficiently via conjugation, resulting in increased colistin resistance in recipient strains [29]. The detection of *mcr* gene variants in both clinical and animal isolates highlights the potential for dissemination among human and animal populations. This raises public health concerns and underscores the need for ongoing surveillance.

Furthermore, the minimum inhibitory concentration (MIC) by the six *E. coli* isolates to colistin (4 µg/mL) revealed that the co-existence of *mcr-1.1* and *mcr-3.5* did not confer an increased resistance to colistin compared to isolates that carry only a single copy of the mcr gene. This finding is similar to a previous report that the co-existence of *mcr-1.1* and *mcr-3.5* might not provide more resistant to colistin [31].

## 3. Material and Methods

### 3.1. Bacterial Strains and Antibiotic Susceptibility Testing

A surveillance study was performed at Queen Sirikit Naval Hospital (QSH) between 2017 and 2018 to identify and characterize MDR bacterial strains from clinical samples [20]. A total of 178 MDR *E. coli* isolates were identified and transferred to the Walter Reed Army Institute of Research–Armed Forces Research Institute of Medical Sciences (WRAIR-AFRIMS) in Bangkok, Thailand, for further analysis. Biochemical identification and antimicrobial susceptibility testing were performed using the BD PhoenixTM 50 with NMIC/ID-4 and NMIC/ID-95 panels (BD Diagnostic Systems, Sparks, MD, USA), following the CLSI guideline 2017 for interpretation [32]. Colistin broth microdilution (BMD) was performed according to the CLSI guideline M07 (11th Edition) for method preparation and M100 (33rd Edition) for antimicrobial susceptibility testing (AST) interpretation [33,34]. *E. coli* ATCC 25922 was used as a quality control strain, with a testing range of 0.5 to 256 µg/mL.

### 3.2. Detection of Extended Spectrum Beta-Lactamase (ESBL), Carbapenemase, and Colistin Resistance Genes

Following the manufacturer’s recommendations, the whole nucleic acid of 178 MDR *E. coli* isolates was extracted using the QIAGEN DNeasy Blood and Tissue Kit (Germantown, MD, USA). Detection of ESBL, carbapenemase, and colistin resistance (*mcr-1* to *mcr*-9) genes was performed using conventional multiplex PCR and real-time PCR as described previously [20].

### 3.3. Short- and Long-Read Whole-Genome Sequencing

Six colistin-resistant *E. coli* isolates were subjected to short-read sequencing on the Illumina MiSeq Benchtop sequencer as described previously (Illumina Inc., San Diego, CA, USA) [21]. Two of the strains carrying both *mcr-1* and *mcr-3* genes were additionally subjected to long-read sequencing on the Pacific Bioscience RSII (PacBio) platform (Pacific Biosciences, Menlo Park, CA, USA) [21].

### 3.4. Analysis of Whole-Genome Sequencing Data

The whole-genome sequencing (WGS) analysis was conducted as described previously [21]. Geneious v10.2.6 (Biomatters, Auckland, New Zealand) was used to perform a comparative genomic analysis. The hybrid de novo assembly of short- and long-read sequence data of AMR-220 and AMR-0361 was conducted by using Unicycler in the Genome Assembly service of the Bacterial and Viral Bioinformatics Resource Center (BV-BRC) server (https://www.bv-brc.org, accessed on 3 October 2023) [35]. In the Genome Assembly Service, the assembled genomes were polished using Pilon (v1.23.) or Racon (v1.4.20.), and the assembled graphs were plotted using Bandage (v0.8.1.). The assembly statistics of the assembled genomes were generated using QUAST (v5.0.2.) and SAMtools (v1.17). The complete genome annotation was performed using RASTtk in the Genome Annotation Service in the BV-BRC server.

Acquired AMR genes were predicted using ResFinder V4.1, the presence of plasmid types was identified with PlasmidFinder 2.1, and the sequence types (STs) of *E. coli* isolates, based on the Achtman scheme, were defined using the MLST 2.0 on the Center for Genomic Epidemiology (CGE) server (http://www.genomicepidemiology.org, accessed on 24 October 2024). Comparative analyses of plasmids carrying the *mcr* gene, with closely related reference plasmids, were generated using the circular genome viewer (CGview v1.1.2), with annotation using Prokka (v1.1.0), and the CARD resistant gene identifier (v1.2.0) on the Proksee server (https://proksee.ca/, accessed on 24 October 2024).

## 4. Conclusions

The colistin-resistant genes *mcr-1* and *mcr-3* were detected in six *E. coli* isolates, with two isolates carrying both genes. While the co-existence of *mcr-1* and *mcr-3* on distinct plasmids is commonly found in animal-derived bacteria, it remains rare in clinical isolates [8,17,28]. We identified co-existing *mcr-1.1* and *mcr-3.5* in 1.1% (2/178) of MDR *E. coli* isolates from Queen Sirikit Naval Hospital, consistent with a 2017 report of clinically isolated *E. coli* carrying *mcr-1.1* and *mcr-3.5* in Thailand [10]. Notably, one of these isolates belonged to ST410, a globally disseminated high-risk clone associated with multidrug resistance. The identification of an ST410 *E. coli* isolate co-harboring two *mcr* genes in a clinical setting is concerning, given that ST410 was recognized for its capability to acquire and disseminate AMR genes [18], suggesting a heightened potential for horizontal gene transfer facilitating the spread of colistin resistance. These findings underscore the critical need for ongoing genomic surveillance and the strengthening of existing infection control strategies to limit the spread of colistin resistance within and beyond clinical settings. Further analysis, including plasmid characterization through conjugation and transformation assays, is crucial for understanding the mechanisms of the co-existence and transmission of these *mcr* genes.

## Figures and Tables

**Table 1:** Antimicrobial susceptibility profile of *mcr*-carrying *E. coli* isolates.

		AMR-0201	AMR-0220 *	AMR-0251	AMR-0354	AMR-0361 *	AMR-0429
Antimicrobial Class	Antimicrobial Agent	Minimum Inhibitory Concentration (μg/mL)					
Carbapenem	Ertapenem	≤0.25 (S)	≤0.25 (S)	≤0.25 (S)	≤0.25 (S)	≤0.25 (S)	≤0.25 (S)
	Imipenem	≤1 (S)	≤1 (S)	≤1 (S)	≤1 (S)	≤1 (S)	≤1 (S)
	Meropenem	≤1 (S)	≤1 (S)	≤1 (S)	≤1 (S)	≤1 (S)	≤1 (S)
Cephem	Cefepime ^4^	>16 (R)	>16 (R)	>16 (R)	>16 (R)	8 (R)	16 (R)
	Ceftazidime ^3^	>16 (R)	>16 (R)	>16 (R)	>16 (R)	8 (R)	16 (R)
	Cephalothin ^1^	>16 (R)	>16 (R)	>16 (R)	>16 (R)	>16 (R)	>16 (R)
	Ceftriaxone ^3^	>16 (R)	>16 (R)	>16 (R)	>16 (R)	>16 (R)	>16 (R)
	Cefuroxime ^2^	>16 (R)	>16 (R)	>16 (R)	>16 (R)	>16 (R)	>16 (R)
	Cefoxitin ^2^	>16 (R)	8 (S)	8 (S)	8 (S)	≤4 (S)	≤4 (S)
β-lactam/β-lactam combination agent	Ampicillin	>16 (R)	>16 (R)	>16 (R)	>16 (R)	>16 (R)	>16 (R)
	Amoxicillin-Clavulanate	>16/8 (R)	8/4 (R)	≤4/2 (R)	8/4 (R)	>16/8 (R)	>16/8 (R)
	Piperacillin-Tazobactam	≤4/4 (S)	≤4/4 (S)	≤4/4 (S)	≤4/4 (S)	≤4/4 (S)	≤4/4 (S)
5-Fluoroquinolone	Ciprofloxacin	>2 (R)	>2 (R)	>2 (R)	≤0.5 (S)	>2 (R)	1 (S)
	Norfloxacin	>8 (R)	>8 (R)	>8 (R)	≤2 (S)	>8 (R)	≤2 (S)
Folate Antagonist	Trimethoprim	>8 (R)	≤1 (S)	>8 (R)	≤1 (S)	>8 (R)	>8 (R)
	Trimethoprim-Sulfamethoxazole	>2/38 (R)	≤0.5/9.5 (S)	>2/38 (R)	≤0.5/9.5 (S)	>2/38 (R)	>2/38 (R)
Aminoglycoside	Gentamicin	>8 (R)	>8 (R)	>8 (R)	≤2 (S)	>8 (R)	>8 (R)
	Tobramycin	8 (I)	8 (I)	8 (I)	≤2 (S)	8 (I)	8 (I)
Nitrofurantoin Polymyxin	Nitrofurantoin	≤16 (S)	≤16 (S)	32 (S)	≤16 (S)	64 (I)	32 (S)
	Colistin ^a^	4 (R)	4 (R)	4 (R)	4 (R)	4 (R)	4 (R)

Note: ^1,2,3,4^ 1st, 2nd, 3rd, and 4th generation of cephalosporin. * *E. coli* isolates that carry both *mcr-1.1* and *mcr-3.5*. ^a^ Colistin MIC by broth microdilution method. S—susceptible; R—resistant; I—intermediate.

**Table 2:** Whole genome sequence analysis reporting sequence types, plasmid replicon types, and identified antimicrobial-resistant genes.

			Aminoglycoside								Beta-Lactam				Polymyxin			Phenicol			Quinolone		Lincosamide	Macrolide			Tetracycline			Trimethoprim			Sulfonamide			Rifampin
																														
Sample ID	MLST	Replicon Type	*aac(3)-IIa*	*aac(3)-IId*	*aadA1*	*aadA2*	*aadA22*	*aph(3)-Ia*	*aph(3)-Ib*	*aph(6)-Id*	*bla* *_CMY-2_*	*bla* *_CTX-M-15_*	*bla* *_CTX-M-55_*	*bla* *_TEM-1_*	*mcr-1.1*	*mcr-3.4*	*mcr-3.5*	*catA2*	*cmlA1*	*floR*	*qnrS1*	*qnrS13*	*lnu(F)*	*mdf(A)*	*mph(A)*	*erm(B)*	*tet(A)*	*tet(B)*	*tet(M)*	*dfrA12*	*dfrA14*	*dfrA17*	*sul1*	*sul2*	*sul3*	*arr-2*
AMR-0201	410	IncFIB, IncI1, IncX4																																		
AMR-0220 *	410	IncFIB, IncI1, IncX4, IncY																																		
AMR-0251	10	IncHI2, IncY																																		
AMR-0354	38	IncFII, IncI1,IncI2																																		
AMR-0361 *	46	IncFIB, IncFII, IncN, IncX4																																		
AMR-0429	617	incFII, IncI1, IncQ1, IncX1, IncFI, IncY																																		

Note: The colored boxes represent the presence of AMR genes corresponding to different antimicrobial classes. * *E. coli* isolates that carry both *mcr-1.1* and *mcr-3.5*.

## Data Availability

The raw genome sequences of Illumina Miseq and PacBio were deposited in the NCBI in FASTQ format, under BioProject PRJNA814829.
